# Eco-evolutionary dynamics of host–microbiome interactions in a natural population of closely related mouse subspecies and their hybrids

**DOI:** 10.1098/rspb.2024.1970

**Published:** 2024-12-18

**Authors:** Susana C. M. Ferreira, Víctor Hugo Jarquín-Díaz, Aimara Planillo, Ľudovít Ďureje, Iva Martincová, Stephanie Kramer-Schadt, Sofia K. Forslund-Startceva, Emanuel Heitlinger

**Affiliations:** ^1^ Division of Computational Systems Biology, Center for Microbiology and Ecological Systems Science, University of Vienna, Djerassipl. 1, Vienna 1030, Austria; ^2^ Department of Molecular Parasitology, Institute for Biology, Humboldt University Berlin (HU). Philippstr. 13 Haus 14, Berlin 10115, Germany; ^3^ Department of Interdisciplinary Life Sciences, Research Institute of Wildlife Ecology, University of Veterinary Medicine Vienna, Savoyenstraße 1, Vienna A-1160, Austria; ^4^ Experimental and Clinical Research Center, Charité–Universitätsmedizin Berlin, Corporate Member of Freie Universität Berlin and Humboldt-Universität zu Berlin, Lindenberger Weg 80, Berlin 13125, Germany; ^5^ Max-Delbrück-Center for Molecular Medicine in the Helmholtz Association (MDC). Robert-Rössle-Str. 10, Berlin 13125, Germany; ^6^ Research Group Ecology and Evolution of Molecular Parasite–Host Interactions, Leibniz Institute for Zoo and Wildlife Research (IZW). Alfred-Kowalke-Straße 17, Berlin 10315, Germany; ^7^ Experimental and Clinical Research Center, a cooperation between the Max-Delbrück-Center for Molecular Medicine in the Helmholtz Association and the Charité–Universitätsmedizin Berlin, Berlin, Germany; ^8^ Department of Ecological Dynamics, Leibniz Institute for Zoo and Wildlife Research (IZW). Alfred-Kowalke-Straße 17, Berlin 10315, Germany; ^9^ Research Facility Studenec, Institute of Vertebrate Biology, Czech Academy of Sciences, Brno, Czech Republic; ^10^ Institute of Ecology, Chair of Planning-related Animal Ecology, Technische Universität Berlin (TUB), Rothenburgstr. 12, Berlin 12165, Germany

**Keywords:** host–microbiome interactions, species barriers, hybridization, microbiome, spatial environment

## Abstract

Closely related host species share similar symbionts, but the effects of host genetic admixture and environmental conditions on these communities remain largely unknown. We investigated the influence of host genetic admixture and environmental factors on the intestinal prokaryotic and eukaryotic communities (fungi, parasites) of two house mouse subspecies (*Mus musculus domesticus* and *M. m. musculus*) and their hybrids in two settings: (i) wild-caught mice from the European hybrid zone and (ii) wild-derived inbred mice in a controlled laboratory environment before and during a community perturbation (infection). In wild-caught mice, environmental factors strongly predicted the overall microbiome composition. Subspecies' genetic distance significantly influenced the overall microbiome composition, and each component (bacteria, parasites and fungi). While hybridization had a weak effect, it significantly impacted fungal composition. We observed similar patterns in wild-derived mice, where genetic distances and hybridization influenced microbiome composition, with fungi being more stable to infection-induced perturbations than other microbiome components. Subspecies' genetic distance has a stronger and consistent effect across microbiome components than differences in expected heterozygosity among hybrids, suggesting that host divergence and host filtering play a key role in microbiome divergence, influenced by environmental factors. Our findings offer new insights into the eco-evolutionary processes shaping host–microbiome interactions.

## 1. Introduction

Microbial biodiversity is recognized as a focus of research with medical and veterinary implications, particularly in relation to host–symbiont associations. The intestinal microbiome comprises taxonomically diverse bacteria, viruses and micro- and macroeukaryotic organisms, which interact with the host either directly (e.g. by activating immunity) or indirectly (e.g. by metabolite production) [1,2]. This complex community contains host-associated residents and environmental-transient components. While bacteria dominate this ecosystem in species number and biomass [3]; other components, such as fungi and eukaryotic parasites also profoundly affect the host [4], particularly on the maturation and activation of the immune system [5,6] and the overall structure of the community [7]. Although not part of the microbiome, diet significantly influences all components of the microbiome [8,9]. Diet depends on the host’s environment, and diet-derived DNA in the intestine can be used to approximate the host’s diet through molecular assessments.

Phylosymbiosis represents the similarities between host-associated microbial communities and their congruence with the evolutionary relatedness of their respective host organisms [10–12]. It arises from different ecological and evolutionary processes occurring simultaneously, particularly deterministic selection by host filtering (i.e. promotion or suppression of taxa mediated by host traits [13]). The diversification of symbionts in parallel with their hosts (co-diversification), microbial random transmission by conspecifics (dispersal) and ecological drift have also been proposed as neutral processes that contribute to phylosymbiosis [14,15]. Phylosymbiosis has been mainly explored within the context of reproductively isolated species [14,16]. Studies of hosts in the early stages of speciation provide a window into the potential divergence of host-associated symbiont communities. In particular, the study of intestinal communities within incipient species or populations with permeable species barriers offers a unique opportunity to decipher the complex relationships between divergent and diverse host genetics and the evolution and ecology of these communities [10,17]. Hybrid zones, especially tension zones stabilized by migration and selection against hybrids [18], serve as a natural laboratory, providing insights into the influence of genetic divergence of parental species and hybrid admixture.

The house mouse hybrid zone (HMHZ) was established at secondary contact between two subspecies of the house mouse, *Mus musculus musculus* (*Mmm*) and *M. m. domesticus* (*Mmd*), which diverged in Asia 0.5 million years ago [19]. This tension zone, approximately 20 km wide [20,21], contains advanced (multi-generation) hybrids between parental subspecies [22]. Hybrids can exhibit transgressive segregation [23], displaying ‘extreme’ traits compared with their parental genotypes, such as increased host resistance to parasites [24,25]. Microbiome composition could be one such a transgressive trait. The HMHZ is ideal for studying the effect of genetic divergence and admixture (hybridization) on the microbiome due to the gradient of genetic variability and environmental heterogeneity [26]. We leveraged a natural population within the HMHZ and wild-derived inbred mice to investigate the effects of subspecies genetics and hybridization on the microbiome, and building on previous studies [17,27], emphasized spatial and temporal ecological determinants. Environmental filtering, represented by spatial distances between hosts can significantly influence microbiome structure, sometimes more than host-associated factors [28,29]. This spatial heterogeneity can result from microbial transmission through host social interactions [30,31] and unevenly distributed environmental factors [32].

Our study aimed to disentangle the relative importance of host filtering, mediated by subspecies’ genetic differentiation and hybridization effects against environmental filtering considering spatial and temporal distribution in shaping the house mouse microbiome. We profiled the components of the intestinal microbiome (bacteria, fungi and parasite communities), and the diet component of wild-caught mice from their natural environment at the HMHZ. We used wild-derived mice in a controlled environment, from both subspecies and their first-generation hybrids to confirm the genetic effects on the intestinal microbiome. Similarly, we profiled the intestinal microbiome before and at the peak of infection with *Eimeria ferrisi*, a common parasite [33] with significant intestinal disruption [34]. Such community perturbation provides a comprehensive understanding of effect robustness. We tested whether intestinal community composition, as a whole or each of its prokaryotic and eukaryotic components, could be differentially defined by host filtering, using as proxies i) host genetic differences between subspecies and (ii) genetic incompatibilities between hybrids leading to aberrant microbiomes. Whether host filtering would lead to (iii) interspecies interactions that shape the microbiome and finally whether (iv) host filtering could be detected independently of environmental filtering, assessed by geographical and temporal distances. Using a combination of wild and wild-derived captive mice, we studied the effect of host genetic admixture and environmental conditions on these communities to test whether host filtering would strongly affect the microbiome components in different ways.

## 2. Material and methods

### (a) Sampling of wild-caught and wild-derived inbred mice

This study utilizes previously published datasets [35,36], which assessed the differential detection and quantification of *Eimeria* spp. using qPCR and single- and multi-amplicon sequencing.

**Figure 1.**
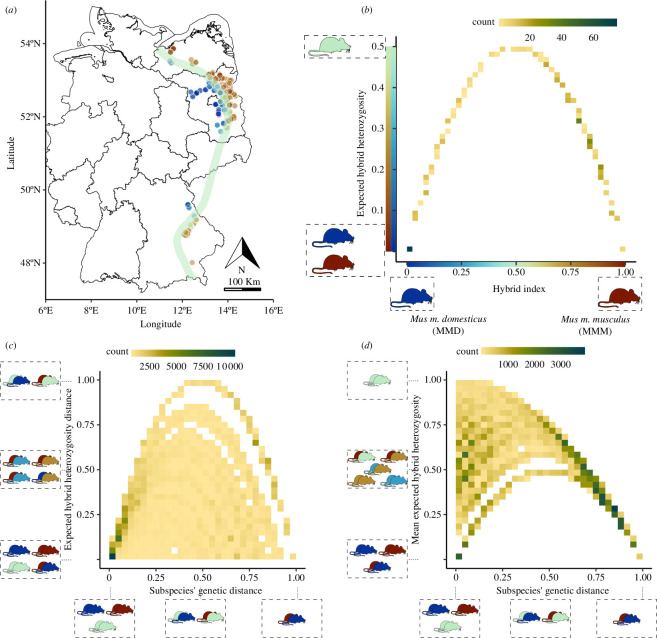
Geographical location of the study area and relationship between genetic measures derived for our analyses. (*a*) Map of the 173 sampling locations within the HMHZ, showing 619 sampled house mice as points, coloured by hybrid index (HI). The HI represents the proportion of *Mmm* alleles genotyped at 14 subspecies-diagnostic loci. The green line marks the HMHZ across Germany. (*b*) Expected hH_e_ represents the degree of admixture in the hybrid genotypes, calculated as $2HI\times \left(1-HI\right)$. Both parental genotypes have an expected hH_e_ of 0, while hybrids with equal admixture between the two subspecies have a maximal hH_e_ of 0.5. (*c*) The relationship between subspecies’ genetic distances (distance between HI values) and differences in hybridization (hH_e_-dist) shows constrained covariance. High hH_e_-dist values are only observed when comparing strongly admixed hybrids with pure parental genotypes. Comparisons between two highly admixed hybrids or two pure subspecies individuals lead to low hH_e_-dist values. (*d*) Mean admixture (mean–hH_e_) measures the average admixture in the compared pair. This genetic term is also constrained with subspecies’ genetic distance. Mouse pairs with large subspecies’ genetic distances tend to have low mean-hH_e_ and thus indicate pure subspecies origin.

The wild mice dataset comprises samples from 672 wild mice captured from 182 locations between 2015 and 2018 (figure 1*a*). Mice within our study area represent two different subspecies (*Mmm* and *Mmd*) or their hybrids, produced through multiple generations of interbreeding. Colon content and tissue samples (muscle and spleen) were collected, preserved in liquid nitrogen and stored at −80°C for microbiome analysis and host genotyping, respectively. Mice were genotyped to estimate the admixture of subspecies genomes, i.e. the hybrid index (HI; table 1), using 14 diagnostic markers as previously described [24,25]. Ninety-nine per cent of the mice had over 10 loci amplified. For three of the mice, fewer loci were amplified, and the hybrid index was imputed by predictive mean matching using the R package ‘mice’ v. 3.16.0, setting meth=‘pmm’ [37].

**Table 1.** Definition of genetic terms.

name	description
HI	value for each mouse calculated as the proportion of *Mmm* alleles in a set of 14 diagnostic markers
expected hybrid heterozygosity (hH_e_)	value ranging between 0 and 1 for each mouse that reflects the degree to which new gene combinations are brought together compared with the pure subspecies, estimated with the expected heterozygosity function: 2HI×(1−HI); parentals have hH_e_ of 0 and hH_e_ is 1 when a hybrid’s genome is estimated to come equally from both subspecies [24]
subspecies’ genetic distance	pairwise difference between HI
expected hybrid heterozygosity distances (hH_e_-dist)	pairwise difference between hH_e_
mean expected hybrid heterozygosity of the comparison (hH_e_-mean)	pairwise sum of hH_e_; measures how admixed the pair is as a whole; as a predictor of community differences, this variable captures whether hybrids have different variance in gut community composition

The wild-derived dataset includes 22 mice of four inbred mouse strains and their F1 hybrids raised under laboratory conditions in the Institute of Vertebrate Biology of the Czech Academy of Science in Studenec (licence: 61974/2017MZE−17214) [38]. Parental genotypes were represented by mice from two allopatric strains: *Mmd* by SCHUNT (*n* = 3 mice) and STRA (*n* = 3 mice) [39], and *Mmm* by BUSNA (*n* = 3 mice) and PWD (*n* = 3 mice) [40]. Two intra-subspecies F1 crosses were also included: *Mmm* PWD × *Mmm* BUSNA (*n* = 3 mice), and *Mmd* SCHUNT x *Mmd* STRA (*n* = 2 mice). Hybrids were represented by two inter-subspecies crosses (*Mmd* STRA × *Mmm* BUSNA, *n* = 2 mice; *Mmd* SCHUNT × *Mmm* PWD, *n* = 3 mice). To ensure a full range of sex combinations, parental strains represent full inbred over 20 generations with maternal and paternal lines alternated. At the animal facility, all mice are kept in Perspex cages at 21–23°C on a 14/10 h light/dark cycle with water and food (pellets, Myška, Pohledští Dvořáci, CZ) available *ad libitum*. Cages are cleaned and bedding is exchanged every 5 days. The neonate mice were co-housed with both parents until the end of weaning. At the age of 20 days, the youngest mice were separated into individual cages. In total, there were eight mice belonging to *Mmd,* nine mice belonging to *Mmm* and five hybrids.

Mice were infected with *E. ferrisi* and samples were collected before infection (day 0) and at the peak of infection (day 6), (experiment licence: 0431/17 issued by Landesamt für Arbeitsschutz, Verbraucherschutz und Gesundheit, Brandenburg). We used the Brandenburg64 isolate of *E. ferrisi*, which had been isolated from the faeces of a wild *Mmd* mouse captured in Brandenburg, Germany. We acclimatized mice to the animal experiment facilities of Humboldt University for at least 1 week before infection. We housed mice in individual cages equipped with tunnels and bedding material for behavioural enrichment and provided them with food and water *ad libitum* during the experiment. We collected three to four faecal pellets from individual mice for DNA extraction.

### (b) DNA extraction, library preparation and sequencing

We extracted genomic DNA from faeces and colon content using the NucleoSpin Soil kit (Macherey-Nagel GmbH & Co. KG, Düren, Germany) following the manufacturer’s protocol with the following modifications: we performed mechanical lysis of the sample in the Precellys 24 high-speed benchtop homogenizer (Bertin Technologies, Aix-en-Provence, France) using two cycles of disruption at 6000 rpm for 30 s, with 15 s delay between cycles. We eluted DNA in 40 µl TE buffer. We assessed the quality and integrity of the DNA using a full-spectrum spectrophotometer (NanoDrop 2000c; Thermo Fisher Scientific, Waltham, MA USA). We quantified the concentrations of double-stranded DNA using a Qubit Fluorometer and the dsDNA BR (broad-range) Assay Kit (Thermo Fisher Scientific). We adjusted DNA extracts to a final concentration of 50 ng µl^−1^ with nuclease-free water (Carl-Roth GmbH+Co. KG) and stored them at −80°C until further processing.

We used faecal DNA preparations for multi-marker amplification using the microfluidics PCR system Fluidigm Access Array 48 × 48 (Fluidigm, San Francisco, California, USA). We randomized sample order and amplified them in parallel with non-template negative controls using a microfluidics PCR. This allows the amplification of multiple fragments (amplicons) for prokaryotic and eukaryotic different hypervariable regions on the ribosomal genes (16S, 18S and 28S), intergenic regions (ITS1 and ITS2), mitochondrial genes (COI and COIII) and apicoplast genes (tRNA and ORF470). The list of primer pairs, target genes and regions are described in electronic supplementary material, additional file 1. We integrated PCR setup library preparation into the amplification procedure according to the protocol for Access Array Barcode Library for Illumina Sequencers (single direction indexing) as described by the manufacturer (Fluidigm, San Francisco, California, USA). The amplicons were quantified using the Qubit fluorometric quantification dsDNA High Sensitivity Kit (Thermo Fisher Scientific, Waltham, USA) and pooled in equimolar concentrations. The final library was purified using Agencourt AMPure XP Reagent beads (Beckman Coulter Life Sciences, Krefeld, Germany). The quality and integrity of the library were confirmed using the Agilent 2200 TapeStation with D1000 ScreenTapes (Agilent Technologies, Santa Clara, California, USA). Sequences were generated at the Berlin Center for Genomics in Biodiversity Research (BeGenDiv) on the Illumina MiSeq platform (Illumina, San Diego, California, USA) using v2 chemistry with 500 cycles.

### (c) Sequencing data processing and decomposition of intestinal community

Data processing and statistical analysis were performed in R v 4.3.1 (R Core Team, 2023). Sequencing reads were filtered, sorted, merged, denoized and chimaeras removed for each run separately and for each amplicon using the R packages dada2 v. 4.3.1 [41] and MultiAmplicon v. 0.1.1 [42]. Contaminants and sequencing errors were removed using the package decontam v. 1.21.0 [43]. We further removed amplicon sequence variants (ASVs) that had less than 1% prevalence, less than 0.005% relative abundance [44] and samples with fewer than 100 reads. Filtering was performed individually for each amplicon in the multi-amplicon datasets, followed by total sum scaling for relative abundances. All amplicon products were collated into one ‘phyloseq’ object [45], resulting in 619 samples in the wild mice dataset and 42 samples in the wild-derived inbred mice dataset.

Taxonomic assignment was performed using the RDP classifier [46] through the dada2 R package [41]. Sequences targeting the 18S, 16S, 28S and ITS rRNA genes were classified against the SILVA 138.1 SSU Ref NR 99, the SILVA 138.1 LSU Ref NR 99 [47], the UNITE [48] databases, respectively. All other sequences from targeted regions without publicly available curated databases were classified against sequences downloaded from NCBI using RESCRIPt [49]. Taxonomy annotation for known parasite genera was refined as developed for coccidians of the genus *Eimeria* in [35] (electronic supplementary material, additional file 2).

Our dataset contained ASVs from different amplicons targeting different marker loci of the same taxon. This resulted in multiple ASVs being assigned to the same taxon, but potentially representing either the same or different variants. To account for this, we merged ASVs likely belonging to the same taxon based on their co-abundance patterns within the same genus. To merge ASVs into cASVs, we constructed a co-abundance network of each genus, consisting of all ASVs annotated within that genus (*n* = 218 co-abundance networks for the wild dataset and *n* = 146 for the laboratory datasets). We calculated Spearman correlations between ASVs and considered only positive correlations (Spearman coefficient >0) that were significant (*p* < 0.01) after multiple testing corrections using the Benjamini–Hochberg method. ASVs with strong co-abundance patterns were clustered together using the ‘cluster_fast_greedy’ function from the R ‘igraph’ package [50]. ASVs within each cluster were merged by summing their relative abundances, resulting in combined ASVs (cASV). The approach, previously applied for the intracellular parasite *Eimeria* spp. [35], was expanded to all other genera in our dataset (electronic supplementary material, additional file 2).

The intestinal community composition was decomposed into four components of cASVs: (i) bacteria including the phyla Firmicutes, Bacteroidota, Deferribacterota, Proteobacteria, Desulfobacterota, Verrucomicrobiota, Actinobacteriota, Campylobacterota, Cyanobacteria, Fusobacteriota, Patescibacteria and unclassified bacteria; (ii) fungi, including the phyla Mucoromycota, Ascomycota and Basidiomycota; (iii) plants (diet), including the phyla Anthophyta, Phragmoplastophyta, Charophyta and Ochrophyta; and (iv) parasites, including the known parasitic genera *Eimeria, Cryptosporidium, Syphacia, Aspiculuris, Mastophorus, Trichuris, Hymenolepis, Tritrichomonas* and order Ascaridida. The models were recapitulated for each component.

### (d) Statistical modelling of community dissimilarity

We calculated the expected hH_e_ (table 1), a variable that captures the nonlinear effect of hybridization [23]. hH_e_ is the expected heterozygosity, specifically for hybrid allele combinations, and represents the degree of admixture in hybrids (figure 1*b*).

Biological communities can be compared using the overall differences between the occurrence and abundance observed in each individual. To quantify the intestinal community variation (β-diversity) among mice, we calculated Jaccard distances (occurrence: presence/absence) with the function ‘distance’ (binary = T) of the R package ‘vegan’ [51]. We repeated the analysis with Aitchison dissimilarity distances (abundance: quantitative), appropriate for use in relative abundances of taxa [52], using the function distance (pseudocount = 1). We transpose both to similarity distances (1 − Jaccard distance; 1 − Aitchison distance).

To test the effects of spatial and temporal distances, species barriers and hybridization on the β-diversity of the intestinal community, we applied Bayesian generalized linear multilevel models using the Markov chain Monte Carlo algorithm No-U-Turn Sampler (NUTS) [53] implemented in Stan through the ‘brms’ R package v. 2.19.0 [54]. The models had intestinal community similarity as the response, and we modelled all possible pairwise distances among mice (excluding comparisons between the same individuals) as previously described [31,55]. We used a multi-membership random effects framework that allows us to account for the individuals in each pairwise comparison (e.g. individuals A and BB). We expressed all predictors as pairwise distances: subspecies’ genetic distance (see table 1; figure 1*c*), distance expected hybrid heterozygosity (hH_e_-dist; see table 1; figure 1*d*), mean expected hybrid heterozygosity (hH_e_-mean; see table 1), spatial distance (Euclidean distances calculated from localities’s spatial coordinates) and temporal distance (difference of time a mouse pair was sampled in years). We included an interaction between subspecies’ genetic distances and hH_e_-dist to allow hH_e_-dist to have different effect strength along the gradient of subspecies’s genetic distances. We scaled all predictors to values ranging from 0 to 1 to allow comparison of standardized estimates of the predictors. We used four Markov chains, with 4 chains, 3000 iterations and 1000 burn-in iterations (warmup) to calibrate the Sampler, and default, uninformative priors. We visually inspected convergence and assessed the relevance of each predictor by analysing R-hat and the 95% credible intervals. A similar model was constructed using the wild-derived inbred mice dataset to test the effects of subspecies’ genetic distance, expected hH_e_-dist and infection status distance (non-infected pairs and infected pairs = 0; non-infected − infected pairs = 1) on the overall microbiome composition and the different components.

### (e) Fungi–bacterial interaction assessment

We investigated the associations between the fungal and bacteria components by modelling the bacterial composition (Jaccard and Aitchison) as a response to the fungal composition (Jaccard and Aitchison, respectively) while controlling for subspecies’ genetic distance, hH_e_dist, hH_e_-mean, spatial distance, and temporal distance. We explored inferred interactions among taxa with prevalence above 5% (present in at least 31 samples).

Co-occurrence networks were created with 171 bacteria, 13 fungi and 6 parasites in a co-occurrence network. Using the R package ‘SpiecEasi’ [56] with the method ‘mb’ neighbourhood selection. We used the extended method for multiple microbial domains (eukaryotes and bacteria) [57]. An optimal lambda value was observed at 0.328, and visualization was performed using the ‘igraph’ R package [50]. Nodes with no edges were excluded from the network for visualisation.

## 3. Results

### (a) Intestinal community profiling in the house mouse hybrid zone

We profiled the intestinal community of 619 wild house mice, captured at 173 localities (figure 1*a*), using a multi-marker approach targeting both prokaryotes and eukaryotes. The final dataset used for the analysis included 588 cASVs (see §2) taxonomically annotated as 106 genera of Bacteria and 77 genera of Eukaryotes, corresponding to eukaryotic parasites (11 cASVs), fungi (65 cASVs) and bacteria (383 cASVs), and also the plant components (45 cASVs) as a proxy of diet.

### (b) Subspecies’ genetic distances and hybridization suggest host filtering as driver in the selection of the intestinal microbiome components in the house mouse hybrid zone

**Figure 2.**
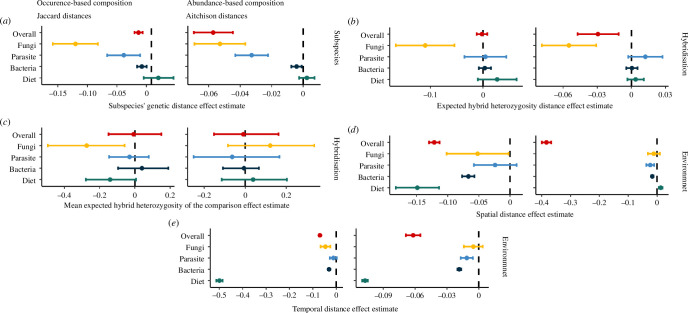
Hybridization leads to aberrant fungal microbiomes. Estimated compositional differences (Jaccard and Aitchison distances) within the overall intestinal communities (overall microbiome, 588 cASVs, red) decomposed community components and plant components (diet) were analysed. Further colours represent the models for the community components fungi (65 cASVs, yellow), parasites (11 cASVs, light blue), bacteria (383 cASVs, dark blue) and diet (45 cASVs, green). The figure shows posterior distributions of the predictor variables: (*a*) subspecies effects measured as subspecies’ genetic distances; (*b*) hybridization effect measured as difference in hybridization (hH_e_-dist); (*c*) hybridization effect measured as mean admixture of the compared pairs (hH_e_-mean); (*d*) environmental effects measured as spatial distances; and (*e*) environmental effects measured as temporal distances in years. While bacteria, fungi and parasites are significantly affected by subspecies differences, only the fungal component is affected by variables related to hybridization. Dots represent the mean effect size, and estimates and bars represent their corresponding credible intervals (level 95%) on intestinal community composition similarity.

First, we investigated the effects of host filtering in the selection of the overall microbiome and each of the community components in wild mice employing two proxies: (i) the subspecies’ genetics and (ii) hybridization (figure 2; electronic supplementary material, tables S1 and S2).

An increase in the subspecies’ genetic distances was associated with differences within the occurrence (Jaccard distances) and abundance (Aitchison distances) of the overall intestinal microbiome (table 2), but also parasites, fungi and bacteria communities independently (figure 2*a*; electronic supplementary material, tables S1 and S2). The similarity within the diet components was not affected by subspecies’ genetic distances. Hybridization had a detectable effect on the overall intestinal microbiome, as the microbiome similarities decreased with increasing hH_e_-dist, but only when abundance-based community distances were assessed (table 2; figure 2*b*). Similarly, differences in hybridization (hH_e_-dist) were significantly associated with reduced fungal community similarities: mice with similar genetic admixture share more similar fungi. As an illustration, two mice from the same parental subspecies (either *Mmm*: HI = 0 or *Mmd*: HI = 1, which form a similarly admixed pair: hH_e_-dist = 0; figure 1*b*) have more similar overall microbiomes and fungi communities than a ‘pure hybrid’ (HI = 0.5) and a parental (HI = 0), which have the biggest differences in admixture (hH_e_-dist = 1, figure 1*b*).

**Table 2.** Distance-based models of the overall intestinal microbiome composition among pairs of individuals (*n* = 191 271) for occurrence-based (Jaccard similarity distances) and abundance-based (Aitchison similarity distances). Shown are the mean estimates of the posterior distribution for each parameter and its associated 95% credible intervals (95% CI), and R-hat value that provides information on the chain convergence (if considerably greater than 1.01, the parameter estimate is not reliable). Bold highlights significant effects (credible intervals do not overlap 0).

	Jaccard similarity distances			Aitchison similarity distances		
	estimate	95% **CI**	Rhat	estimate	95% **CI**	Rhat
intercept	−1.263	−1.341: −1.150	1.25	−2.008	−2.105: −1.919	1.04
spatial distances	**−0.122**	**−0.130: −0.113**	**1.00**	**−0.383**	**−0.399: −0.367**	**1.00**
subspecies’ genetic distance (divergence)	**−0.022**	**−0.029: −0.015**	**1.00**	**−0.057**	**−0.069: −0.045**	**1.00**
hH_e_-dist (admixture)	−0.002	−0.012: 0.008	1.00	**−0.029**	**−0.047: −0.011**	**1.00**
hH_e_-mean (admixture)	−0.015	−0.212: 0.141	1.49	0.009	−0.152: 0.162	1.03
temporal distance	**−0.070**	**−0.074: −0.066**	**1.00**	**−0.062**	**−0.069: −0.055**	**1.00**
subspecies’ genetic distance: hH_e_-dist	0.008	−0.012: 0.028	1.00	**0.047**	**0.010: 0.082**	**1.00**

We observed that occurrence-based fungal community similarity decreased with increasing mean admixture (hH_e_-mean), indicating an increase in variance in the fungal community composition with hybridization. Comparisons among ‘pure hybrids’ (highest hH_e_-mean; figure 1*d*) showed higher variance within their fungal compositions compared with pairs of pure parentals (lowest hH_e_-mean). In contrast, mean admixture (hH_e_-mean) did not significantly affect abundance- or occurrence-based community similarities among the overall microbiome, parasite and bacteria communities, and neither among the diet components (table 2; figure 2*e*,*f*). Thus, we did not detect effects of hybridization on the variance of these microbiome components and diet compositions.

We found a positive interaction effect between hybridization differences (hH_e_-dist) and subspecies’ genetic distance on the overall microbiome and fungal composition. This suggests that the strength effect of the hybridization decreases as subspecies genetic distance increases (electronic supplementary material, figure S3). In natural populations, we observed that genetic effects associated with both house mice subspecies and hybridization strongly influence different components of the microbiome, suggesting that host filtering processes linked to host genetics play an important role in shaping different communities within the microbiome, particularly for fungi communities.

### (c) Environmental filtering driven by geography determine the bacterial composition within the intestinal microbiome in the house mouse hybrid zone

To determine environmental filtering for selection of the microbial composition structure in natural populations of mice, we use geographical and temporal distances as predictors of the microbial community. Overall, intestinal communities were more similar when mice were captured from geographically closer sites and within shorter timeframes (table 2; figure 2*d*,*e*). Spatial proximity was the strongest predictor of overall microbiome composition similarities among pairs of mice (table 2; figure 2*d*). We also observed that microbiome similarity decreased with increased temporally distances between sampling years (table 2; figure 2*e*). For bacteria, both occurrence- and abundance-based composition showed decreased similarity with greater spatial distances. Similarly, fungi and diet-derived occurrence-based, as well as parasite abundance-based compositions, also decreased in similarity with increased spatial distances (figure 2*d*). Increased temporal distances reduced the similarity of bacteria and diet composition, as well as fungi occurrence- and parasite abundance-based compositions (figure 2*e*). Suggesting an additional selection driven by the environment, which is independent from the host filtering effect.

### (d) Fungi composition predicts bacterial composition in the house mouse hybrid zone

**Figure 3.**
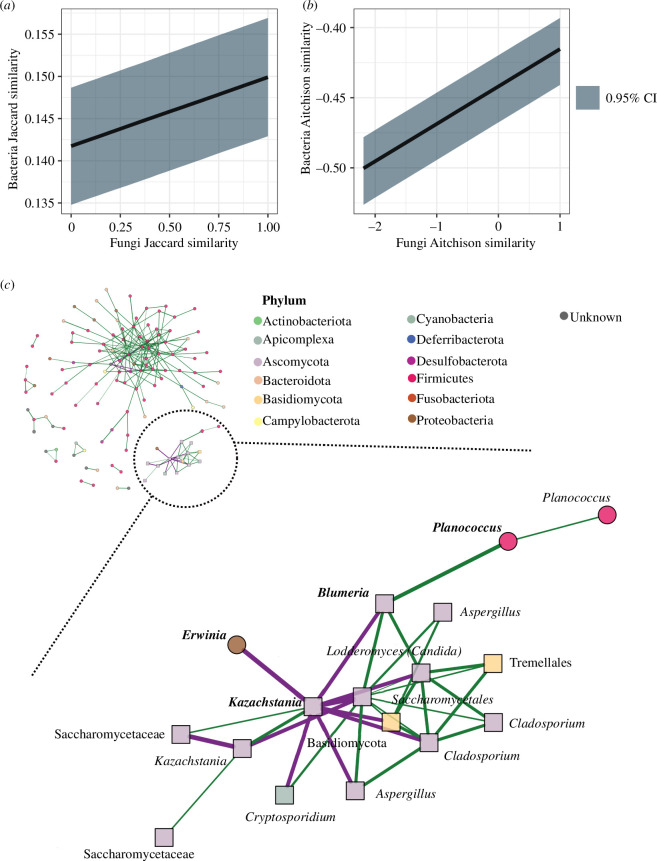
The similarity of the bacterial community increases with fungal community similarity. Conditional effect plots with 95% credible intervals (CI), predicted from model fit (see §2) using (*a*) occurrence-based (Jaccard distances) and (*b*) abundance-based (Aitchison distances) composition. Black line is the average predicted relationship between bacteria similarity and fungi similarity and the shaded area is the 95% credible interval. This effect is independent of subspecies’ genetic, temporal and spatial effects. (*c*) Co-occurrence network of bacteria, eukaryotic parasites and fungi with a prevalence of at least 5% (31 samples). The segment of the network containing fungi-bacteria interactions was enlarged. Nodes correspond with cASVs, and colours correspond to the respective phyla. Circles represent bacteria and squares represent eukaryotes. Labels are the lowest taxonomic annotation for each cASV. Interacting fungi and bacteria are in bold. Edges are predicted interactions; green edges are positive and purple edges are negative. Association strength is represented by the edge thickness.

Effects on different components of the intestinal community may not be independent, so we tested whether differences in the fungal community predict differences in the bacterial community. We did this while controlling for subspecies’ genetic, temporal and spatial effects and found that the fungal community composition predicted bacterial composition in both occurrence- (posterior mean estimate of 0.008, CI from 0.007 to 0.009) and abundance-based measures (posterior mean estimate of 0.027, CI from 0.026 to 0.028; figure 3*a*,*b*; electronic supplementary material, table S3). To explore whether the selection of specific fungi would impact other bacteria, we looked for direct associations between taxa. We used a co-occurrence network and found 239 significant associations (edges) within 127 taxa (nodes) (figure 3*c*). We found only two associations between bacteria and fungi, as the bacteria *Erwinia* and *Planococcus* were associated with the fungi *Kazachstania* and *Blumeria* and one edge between the parasite *Cryptosporidium* and the fungi *Kazachstania,* and a member of the family Saccharomycetales. These results indicate that host filtering on fungal communities appears to be independent of fungi-bacteria interactions, with only a few direct interactions being affected.

### (e) Host filtering driven by subspecies and hybridization affects the intestinal community independently of the environment and community perturbation under experimental setups

We tested whether host filtering driven by subspecies differences is reflected in the intestinal microbiome composition controlling for environmental effects but inducing a microbiome perturbation through a parasite infection. Using a controlled laboratory setting, we profiled 22 ‘wild-derived’ inbred house mice before and at the peak of *E. ferrisi* infection (day 0 and day 6, respectively), applying the same multi-marker approach. From 597 ASVs across 12 amplicons, we obtained 318 cASVs. We taxonomically annotated 36 genera of Eukaryotes and 81 genera of Bacteria, and performed further analysis of the cASVs communities, as described for the wild mice (§2). Of the cASVs, 207 were bacteria, 29 were Fungi, six were parasites and 30 were diet-related plants.

**Figure 4.**
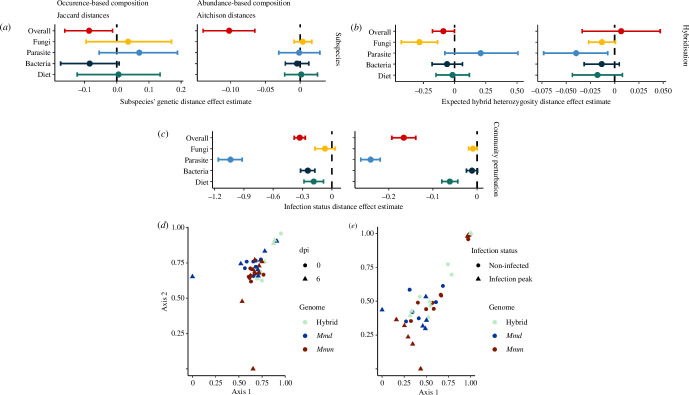
The fungal community of wild-derived inbred mice is shaped by hybridization effects. The compositional differences (Jaccard and Aitchison distances) between whole intestinal communities (full model, 318 cASVs, red) and decomposed community components were analysed for 22 mice before *Eimeria ferrisi* infection (day 0) and at the peak of infection (day 6). Further colours represent the models for the community components fungi (29 cASVs, yellow), parasites (6 cASVs, light blue), bacteria (207 cASVs, dark blue) and plants (30 cASVs, green). The figure shows posterior distributions of the predictor variables: (*a*) subspecies effects measured as subspecies’ genetic distances; (*b*) hybridization effect measured as differences in hybridization (hH_e_-dist); (*c*) community perturbation (experimental infection with *E. ferrisi*) measured as infection status distances. Only the overall intestinal microbiome is significantly affected by subspecies differences, whereas only the fungal component is affected by hybridization. Dots represent the mean effect size estimates and bars represent their corresponding credible intervals (level 95%) on intestinal community composition similarity. The model also accounted for infection status. While *Eimeria* infection shows an overall perturbation of the intestinal microbiome, this was not reflected in the fungal component, specifically (electronic supplementary material, table S4). Principal coordinate analysis of Jaccard distances for (*d*) the overall intestinal community, (*e*) the fungi community. Point shape indicates infection state and colour indicates mice genotype.

Our findings confirm those observed in the wild mice: (i) the similarity of the overall intestinal community decreased as subspecies genetic distances increased (figure 4*a*,*d*; electronic supplementary material, table S4), and (ii) both fungi occurrence-based composition and parasite abundance-based composition decreased with increasing hybridization differences (hH_e_-dist) (figure 4*b*,*e*; electronic supplementary material, table S3). Furthermore, experimental infection with *E. ferrisi* strongly perturbates the overall intestinal community, particularly affecting the bacteria and parasite communities and diet components (figure 4*c*). These results highlight that host genetic differences, even under controlled environmental conditions and perturbation, play a significant role in shaping microbial community composition, suggesting that in house mice microbiomes are under deterministic selection through host filtering mechanisms.

## 4. Discussion

The evolutionary history of hosts influences the similarity of their microbiomes, suggesting that differences in intestinal community are established during the speciation process [11,12]. In house mice particularly, both subspecies divergence and hybridization have been shown to affect the bacterial microbiome [17] and specific eukaryotic parasites [24,25,58]. Our results show that genetic differentiation between the two subspecies of house mouse significantly affects the microbial composition of the intestinal community. This was shown in natural populations, taking into account spatial and temporal variation, complementing recent independent work [27]. We extended these findings to a laboratory setting where subspecies genetic differentiation was detectable in wild-derived inbred mice, both with and without community perturbation. Although the overall effect of hybridization was small, it significantly influenced the fungal community: hybrids exhibited a distinct fungal composition compared with parental mice in both natural populations and wild-derived inbred mice. Our study provides new insights into non-neutral processes and highlights the role of host genetics and environmental factors in shaping microbiome and symbiont communities.

The neutral assembly model suggests that community composition is driven solely by stochastic processes, including random dispersal, speciation, extinction and ecological drift [59,60]. In contrast, non-neutral assembly models propose that selective pressures such as niche, diet and host genetics shape microbial communities [15]. Our results show that genetic differences between house mouse subspecies are associated with variation in the intestinal microbial community under both natural (uncontrolled) environmental and laboratory (controlled) conditions. Our findings are consistent with previous work showing the strong effects of host phylogenetic relationship in wild and captive animals from several rodent species [61]. We suggest that other deterministic processes, in particular host filtering, explain our results, where host traits promote or suppress certain taxa in the community [13].

Host genetic differences may influence the intestinal environment through mechanisms such as genotype-specific immune responses, metabolism and behaviour [62–64]. These mechanisms shape both the abiotic and biotic host environment of each subspecies, creating distinctive niches that support specific microbial communities [65]. Beyond the genetic effect, we need to acknowledge that both parental subspecies and hybrids may be exposed to different microbial pools, adding a layer of complexity that could confound the genetic effects. To disentangle environmental and genetic effects, we used wild-derived mice bred under controlled laboratory conditions, using strains from different locations and different genetic backgrounds. We showed that these wild-derived mice also have species-specific microbiome compositions: strains from different subspecies have greater differences in overall microbial composition. Our results are consistent with the observed differences in the intestinal bacterial community between subspecies of captive mice bred under a common garden approach [26], and support the idea of host filtering as a predictor of microbial composition. This present-day association of intestinal communities with their hosts does not necessarily indicate co-adaptation or its relevance in the speciation process [13]. During the early divergence of house mouse subspecies, ecological drift and changes in the host’s environment [30] may have been the predominant force of community divergence. Our results confirm that host genetic differentiation has an effect on the maintenance of distinct microbial communities.

Hybridization effects can indicate species barriers, as genetic incompatibilities might induce transgressive traits that negatively affect hybrid fitness [66]. A previous study showed a differentiation in the intestinal bacterial community between wild-derived second-generation (F2) hybrids and pure subspecies in a laboratory setting and in a wild population [17]. In contrast to this study, we could not confirm the hybridization effect on the bacterial community considering hybridization as a nonlinear gradient ranging between the two subspecies, although we found changes in the fungal microbiome associated with hybridization, as did a recent study [27]. Interestingly, we observed that the strength of the hybridization effect varies along the gradient of subspecies’s genetic distances, suggesting that the extent of these effects on the fungal microbiome may depend on the specific levels of hybridization between the subspecies. We also detected an increase in the variance of the fungal occurrence, indicating that aberrant fungal microbiome compositions are found among pairs of hybrids within natural populations.

We confirmed that the aberrant fungal composition in hybrids was independent from the environmental conditions. The first-generation hybrids of wild-derived mice also had a fungal microbiome composition distinct from that of parentals in the laboratory. Hybrids at the animal facility are exposed to a microbial pool from both subspecies’ genotypes in early life, which could result in compositional differences due to foundational effects from pioneer taxa transmitted from both parents [67]. It is possible that these differences could persist to adulthood and contribute to the patterns observed in the laboratory setting. Community perturbations can change species composition within a community [68] and reduce founder effects [69]. We exposed wild-derived mice to a community perturbation through infection with *E. ferrisi* and tested the stability of the intestinal microbiome. Independently from the community perturbation, fungal symbionts were affected by hybridization. Although we cannot rule out founder effects, our results suggest that these effects are unlikely to define fungal communities in hybrid mice and hybridization has a greater influence.

In addition to the resilience of fungal communities to community perturbations, we also observed that fungal symbionts were less affected by external environmental filtering in the wild compared with other microbiome components. These observations suggest that fungal intestinal communities are more stable in response to changes in the external environment (e.g. geography) and internal short-term perturbations (e.g. parasite infections). However, they are still subject to other significant continuous selection pressures within the internal host environment, as indicated by our results on genetic distances and hybridization. Host filtering, mediated by aberrant immune-related gene expression leading to higher inflammation in the gut of hybrids [17] imposes strong selective pressure on both the prokaryotic and eukaryotic components of the microbiome. Nevertheless, our results suggest that host filtering specifically selects the composition for fungal communities, despite their resilience to environmental filtering. The ‘unfavourable’ conditions inside the host, while impacting the overall fungal community in the long term, may favour other specific microbial populations that are metabolically independent and resilient to continuous stress, and can self-sustain in the absence of the ecological services associated with a homeostatic environment [70].

Bacteria–fungi interactions are widespread in various communities, including intestinal ones [7,71]. The fungal component is a primary source of secondary metabolites, such as natural antimicrobial compounds, which could affect the presence and abundance of bacteria and shape microbial communities at taxon level, influencing the gene content like virulence factors and antimicrobial resistance genes (ARGs) [6,72]. We found that mice with similar fungal compositions also had similar bacterial compositions. However, we detected very few bacteria–fungi associations. The current study cannot define the mechanistic interactions between these two components that lead to few direct associations. Transmission, colonization and host filtering of the fungal component of the microbiome are a frontier for further research both in the laboratory and in the natural environment.

Our findings indicate that host genetic differences impact the intestinal symbiont communities in the two house mouse subspecies and reflect host–symbiont interactions. We observed that while fungi communities are the most affected component of the microbiome by hybridization, both in natural or laboratory conditions, they are highly resilient to spatial or short-term community perturbations. Aberrant effects of hybridization on symbionts could suggest the involvement of symbiont interactions earlier in the process of speciation. In the wild, host filtering (e.g. immune regulation and control of susceptibility or due to slight divergence of the species’ environmental niche) cannot be completely distinguished from environmental filtering (geographical and temporal effects). However, our laboratory results under more environmentally controlled conditions suggest direct host-mediated filtering of the microbiome and stability of hybrid-differences, especially in fungi against community perturbation. Future studies should combine immune measures and detailed environmental characterisation (e.g. microclimate, agricultural practices or general land-use) to disentangle these contributions. Experimental set-ups of wild-derived parentals and hybrids should have a particular focus on fungal communities, while also playing attention to interactions between taxa within the microbiome.

## Data Availability

The datasets and a fixed version of the scripts supporting the conclusions of this article are available at [73]. All sequencing raw data can be accessed through the BioProjects PRJNA948184 and PRJNA912123 in the NCBI SRA. Supplementary material is available online [74].
